# Phosphoproteome Profiling Reveals Circadian Clock Regulation of Posttranslational Modifications in the Murine Hippocampus

**DOI:** 10.3389/fneur.2017.00110

**Published:** 2017-03-22

**Authors:** Cheng-Kang Chiang, Bo Xu, Neel Mehta, Janice Mayne, Warren Y. L. Sun, Kai Cheng, Zhibin Ning, Jing Dong, Hanfa Zou, Hai-Ying Mary Cheng, Daniel Figeys

**Affiliations:** ^1^Faculty of Medicine, Department of Biochemistry, Microbiology and Immunology, Ottawa Institute of Systems Biology, University of Ottawa, Ottawa, ON, Canada; ^2^Department of Chemistry, National Dong Hwa University, Shoufeng, Hualien, Taiwan; ^3^Department of Biology, University of Toronto Mississauga, Mississauga, ON, Canada; ^4^Key Laboratory of Separation Science for Analytical Chemistry, Dalian Institute of Chemical Physics, Chinese Academy of Sciences, Dalian, China; ^5^Department of Chemistry and Biomolecular Sciences, University of Ottawa, Ottawa, ON, Canada; ^6^Canadian Institute for Advanced Research, Toronto, ON, Canada

**Keywords:** hippocampus, circadian rhythm, quantitative proteome and phosphoproteome analysis, phosphorylation, kinase–substrate relations

## Abstract

The circadian clock is an endogenous oscillator that drives daily rhythms in physiology, behavior, and gene expression. The underlying mechanisms of circadian timekeeping are cell-autonomous and involve oscillatory expression of core clock genes that is driven by interconnecting transcription–translation feedback loops (TTFLs). Circadian clock TTFLs are further regulated by posttranslational modifications, in particular, phosphorylation. The hippocampus plays an important role in spatial memory and the conversion of short- to long-term memory. Several studies have reported the presence of a peripheral oscillator in the hippocampus and have highlighted the importance of circadian regulation in memory formation. Given the general importance of phosphorylation in circadian clock regulation, we performed global quantitative proteome and phosphoproteome analyses of the murine hippocampus across the circadian cycle, applying spiked-in labeled reference and high accuracy mass spectrometry (MS). Of the 3,052 proteins and 2,868 phosphosites on 1,368 proteins that were accurately quantified, 1.7% of proteins and 5.2% of phosphorylation events exhibited time-of-day-dependent expression profiles. The majority of circadian phosphopeptides displayed abrupt fluctuations at mid-to-late day without underlying rhythms of protein abundance. Bioinformatic analysis of cyclic phosphorylation events revealed their diverse distribution in different biological pathways, most notably, cytoskeletal organization and neuronal morphogenesis. This study provides the first large-scale, quantitative MS analysis of the circadian phosphoproteome and proteome of the murine hippocampus and highlights the significance of rhythmic regulation at the posttranslational level in this peripheral oscillator. In addition to providing molecular insights into the hippocampal circadian clock, our results will assist in the understanding of genetic factors that underlie rhythms-associated pathological states of the hippocampus.

## Introduction

Many behavioral and physiological processes exhibit daily fluctuations or circadian rhythms, which are governed by an intrinsic timekeeping mechanism. The circadian system ensures that the timing of these processes is optimal with respect to other ongoing internal events as well as to the external environment. In mammals, a central pacemaker that is situated in the suprachiasmatic nucleus (SCN) of the brain coordinates rhythms in peripheral tissues (1–3). Circadian timekeeping, be it central or peripheral, is a cell-autonomous phenomenon that is based on interconnecting transcription–translation feedback loops. To date, several large-scale proteomic studies of circadian regulation within the SCN (4, 5) as well as in peripheral tissues such as the heart (6) and liver (7, 8) have been conducted to understand clock-controlled mechanisms at the protein level. Chiang et al. (4) reported that temporal regulation of mitochondrial oxidative phosphorylation, as well as posttranscriptional regulation, plays an essential role in the SCN. Robles et al. (7) and Mauvoisin et al. (8) showed that metabolic and physiological functions in the liver are under circadian control at the transcriptional, posttranscriptional, and posttranslational levels.

In addition to the liver, several studies have reported the presence of circadian oscillations within the hippocampus, a region in the brain associated with the conversion of short- to long-term memory (9, 10). Schaaf et al. (11) and Wang et al. (12) found that the expression of brain-derived neurotrophic factor and Period2 (Per2), respectively, are circadian in the rodent hippocampus. Furthermore, genetic ablation of Per2 or Period1 (Per1) in mice leads to disruptions in hippocampal-dependent trace fear conditioning (12) and spatial memory performance (13). Given the pivotal role of cAMP-responsive element-binding protein (CREB) in memory processing (14–16), the observed rhythm in CREB phosphorylation in the hippocampus (13), but also in the SCN (14–16), provides a molecular mechanism by which the circadian clock may control the formation of long-term memories. Overall, the evidence of circadian rhythmicity within the hippocampus has provided an intriguing basis for understanding memory formation.

To understand how hippocampal function is impacted by the circadian clock, we sought to define the circadian proteome and phosphoproteome of the murine hippocampus, since phosphorylation is a major posttranslational modification (PTM) that regulates protein function. Using super-stable isotope labeling by amino acids in cell culture (super-SILAC) (17) and high accuracy mass spectrometry (MS), we identified in our unbiased screen a total of 4,953 unique proteins and 9,478 phosphorylation events. Out of those, there were 149 phosphorylation events that displayed circadian profiles of expression at the posttranslational level only. Bioinformatic analyses revealed that the circadian phosphoproteome peaked in the mid-to-late day and that those rhythmic phosphoproteins were preferably involved in diverse biological functions such as synaptic processes and cytoskeletal organization.

## Materials and Methods

### Ethics Statement

All animal experiments were conducted at the University of Toronto at Mississauga and were approved by the local animal care committee in compliance with institutional guidelines and the Canadian Council on Animal Care. Male C57BL/6J mice purchased from the Jackson Laboratory (Bar Harbor, ME, USA) were utilized for all experiments. Mice were group-housed in polycarbonate cages and given *ad libitum* access to rodent chow and water throughout the study.

### Tissue Collection

Thirty male C57BL6/J mice, aged 8–12 weeks, were stably entrained for a minimum of 2 weeks to a 12-h light:12-h dark (LD) schedule (light intensity during the light phase was 200 lux) prior to transfer to complete darkness (DD) for two full cycles. Dark adaptation was achieved by placing cages into light-tight ventilated cabinets. On day 3 of DD, five mice were sacrificed at each time point corresponding to circadian time (CT) 2, 6, 10, 14, 18, and 22, where CT was defined by the Zeitgeber time of the previous LD schedule. Mice were killed by cervical dislocation and decapitated under dim red light, and eyes were covered with black electrical tape. Subsequently, whole hippocampal tissues were dissected, immediately flash-frozen in liquid nitrogen, and stored at −80°C until further processing.

### Proteomic Analysis of Hippocampal Tissues Using Super-SILAC-Based Quantitative MS

To isotopically label murine cells, five cell lines, including Neuro-2a (neuroblastoma), AtT-20 (pituitary) acquired from ATCC (Manassas, VA, USA), mHypoE-N38, mHypoA-2/21 (CLU-181), and mHypoA-2/28 (CLU-188) (hypothalamus) acquired from Cedarlane Laboratories (Toronto, ON, Canada) were individually cultured in SILAC media at 37°C in a 5% CO_2_ humidified incubator. For the SILAC media, customized DMEM by AthenaES (Baltimore, MD, USA) in which the natural lysine and arginine were replaced by heavy isotope-labeled amino acids, ^13^C_6_^15^N_4_ l-arginine (Arg 10) and ^13^C_6_^15^N_2_ l-lysine (Lys 8) was supplemented with 10% (v/v) dialyzed FBS (GIBCO-Invitrogen; Burlington, ON, Canada), 1 mM sodium pyruvate (GIBCO-Invitrogen), and 28 μg/mL gentamicin (GIBCO-Invitrogen). Complete (>98%) incorporation of the isotopically labeled amino acids into cellular proteins was achieved after at least 10 cell doublings in SILAC media.

Hippocampal tissues were homogenized in 300 μL of lysis buffer (8 M urea, 50 mM Tris–HCl (pH 7.5)), 100 mM DTT, 4% (v/v) SDS, 1 mM sodium orthovanadate supplemented with proteinase inhibitor cocktail (Roche, Mississauga, ON, Canada) and phosphoSTOP phosphatase inhibitor cocktail (Roche) with a pellet pestle and sonicated three times with 10 s pulses each (>30 s) on ice between each pulse. Protein concentrations were determined using the Bio-Rad DC Protein Assay. Hippocampal lysates (1 mg) and super SILAC-labeled cell lysates (0.2 mg from each of Neuro-2a, AtT-20, mHypoE-N38, CLU-181, and CLU-188 cells) were mixed at a 1:1 weight ratio, and SDS in solution was removed by an overnight incubation at −20°C in five volumes of ice-cold precipitation buffer [acetone/ethanol/acetic acid (v/v/v) = 50/50/0.1]. The precipitated proteins were washed twice with ice-cold acetone, and the protein pellets were redissolved in 50 mM NH_4_HCO_3_ solution containing 8M urea. For in-solution trypsin digestion, 1.2 mg of proteins in each sample was reduced with 5 mM DTT (Sigma, St. Louis, MO, USA) at 60°C for 1 h and alkylated with 10 mM iodoacetamide (Sigma) in the dark (40 min at room temperature). Each sample was diluted in fivefold volume of 50 mM NH_4_HCO_3_ (pH 8.5) solution to reduce the urea concentration to <2 M and digested overnight with TPCK-treated trypsin (Worthington, Lakewood, NJ, USA) at an enzyme-to-protein ratio of 1:25 (w/w). For proteomic analysis, 0.1 mg of resulting peptides were fractionated through an in-house constructed strong cation exchange (SCX) column with five pH fractions (pH 4.0, 6.0, 8.0, 10.0, and 12.0) followed by desalting with in-house C18 desalting cartridges and dried in a speed-vac prior to LC-MS analysis. The remaining 1.1 mg of tryptic peptides were desalted by SepPak C18 cartridges (Waters, Mississauga, ON, Canada), dried, and SCX fractionated into four fractions (pH 4.0, 6.0, 8.0, and 12.0) prior to phosphoproteome enrichment.

### Hippocampal Phosphoproteome Enrichment by Ti^4+^-IMAC Chromatography

Ti^4+^-IMAC beads preparation and the phosphopeptide enrichment procedure were performed as described previously (18). Samples were resuspended in 1 mL loading buffer (80% (v/v) acetonitrile (ACN) and 6% (v/v) TFA) with 300 μL of Ti^4+^-IMAC bead slurry (10 mg beads in 1 mL of loading buffer) for 30 min at 4°C. After centrifugation (16,000 × *g*, 10 min), beads were washed with 200 μL of washing buffer 1 [50% (v/v) ACN, 6% (v/v) TFA, and 200 mM NaCl], followed by two washes with 200 μL of washing buffer 2 [30% (v/v) ACN and 0.1% (v/v) TFA]. Phosphopeptides were eluted from the beads using 200 μL of 10% (v/v) ammonia solution for 15 min and sonicated for another 15 min at 4°C. After centrifugation, the retrieved supernatants were collected, acidified with 10% (v/v) TFA, desalted with in-house C18 desalting cartridges, and dried in a speed-vac prior to LC-MS analysis.

### LC-MS Analyses

All resulting peptide fractions were reconstituted in 20 μL of 0.1% (v/v) FA and 4 μL of each sample was analyzed by online reverse-phase LC-MS/MS platform consisting of an Eksigent NanoLC-Ultra 2D plus system (AB SCIEX) coupled with a Q Exactive Hybrid Quadrupole-Orbitrap mass spectrometer (Thermo Fisher Scientific, San Jose, CA, USA) *via* a nano-electrospray source. Peptide mixtures were separated by reverse phase chromatography using a home-packed ReproSil-Pur C18-AQ column (75 μm internal diameter × 15 cm, 1.9 μm, 200 Å pore size; Dr. Maisch GmbH, Ammerbuch, Germany) in 2 h LC gradient of 2–80% buffer B [ACN in 0.1% (v/v) FA] at a flow rate of 300 nL/min. The Q Exactive instrument was operated in the data-dependent mode to simultaneously measure survey scan MS spectra (from *m/z* 400–2,000) in the Orbitrap analyzer at resolution *R* = 70,000. Up to the 12 most intense peaks with charge state ≥2 and above a signal threshold of 500 counts were selected for fragmentation in the ion trap *via* higher-energy collisional dissociation. System controlling and data collection were carried out by Xcalibur software version 2.2 (Thermo Scientific).

### Mass-Spectrometry Database Search and Bioinformatic Analysis

Mass spectrometry data from the hippocampal proteome and phosphoproteome were analyzed and quantified with MaxQuant (version 1.3.0.5) using Andromeda as the search engine against the UniProt (release 2014_04) database restricted to Mouse (*Mus musculus*) taxonomy concatenated with decoy reversed sequences. The precursor ion mass tolerance was 6 ppm and fragment mass deviation was 0.5 Da for MS/MS spectra. The search included variable modifications of methionine oxidation, N-terminal acetylation, Ser/Thr/Tyr phosphorylation, and fixed modification of cysteine carbamidomethylation. Trypsin/P (cleavage after Lysine and Arginine, including Lysine–Proline and Arginine–Proline) was set as the cleavage specificity with two missed cleavages. The false discovery rate (FDR) cutoffs for peptide and protein identification were both set to 0.01 and the minimum peptide length was set to 7. Identification across different replicates and adjacent fractions were achieved by enabling match between runs option within a time window of 2 min. Default settings were used for all the other parameters in MaxQuant. The proteingroup file from hippocampal proteome and Phospho (STY) Sites file from the phosphoproteome were imported into Perseus (version 1.5.2.4) for the analysis. The raw proteomic and phosphoproteomic datasets (4,953 proteins and 9,478 phosphosites, respectively) were filtered to include only proteins/phosphosites with quantification values in a minimum of 15 of 30 MS measurements (or 30 independent hippocampal samples), resulting in a stringently quantified dataset of 3,052 proteins and 2,868 phosphosites, respectively. Hierarchical clustering analysis, using the median value of logarithmized values for the normalized L/H ratio of each protein and phosphopeptide profile, was performed after *z*-score normalization of the data within Euclidean distances.

To identify the subset of 24-h rhythmic proteins and phosphopeptides, JTK_CYCLE algorithm (19) was used on the hippocampal proteomic (3,052 proteins) or the phosphoproteomic (2,868 phosphorylation events) dataset under R language. Prior to JTK_CYCLE analysis of those two datasets, any missing values (i.e., not detected by MS) were replaced with 0 and the minimum values observed in each screen (4), respectively. Only if both replacement methods showed a significant profile [*p*-Values (ADJ.P) less than 0.05] were the corresponding proteins/phosphopeptides classified as displaying a circadian rhythm. To find the 8- or 12-h rhythmic proteins and phosphoproteins within the 3,052-protein and 2,868-phosphoprotein datasets, another JTK_CYCLE analysis was separately performed with period lengths set at 8- and 12-h.

Unsupervised clustering analysis (fuzzy *c*-means) of the temporal profiles of cycling phosphopeptides was performed using the Mfuzz package (20) in R. The gene ontology (GO) annotation and pathway enrichment analysis of circadian phosphoproteins were implemented using the DAVID (21). The ingenuity pathway analysis (IPA) software (version 7.5) was utilized to analyze the biological functions, protein–protein interactions, and signaling pathway annotations of the rhythmically expressed proteins and phosphoproteins. Motif analysis was performed using iceLogo (22), scoring by percent difference with a significance threshold of 0.05 for a sequence window of 15 amino acids surrounding the phosphorylated residues on hippocampal circadian phosphopeptides. Protein interaction network analysis of the cycling phosphoproteome was performed with the STRING (23) using medium to high confidence (0.5–0.7) and with co-expression and experiments as active prediction methods. iGPS 1.0 (24) was used to predict possible site-specific kinase–substrate relations (ssKSRs) between putative protein kinases (PKs) and circadian hippocampal phosphopeptides using a high threshold.

The MS proteomics and phosphoproteomics data have been deposited to the ProteomeXchange Consortium (http://www.proteomexchange.org) *via* the PRIDE partner repository (25) with the dataset identifier PXD005668 and PXD005669, respectively.

## Results

### Super-SILAC-Based Quantitative Proteomic and Phosphoproteomic Analysis of the Murine Hippocampus

**Figure 1:**
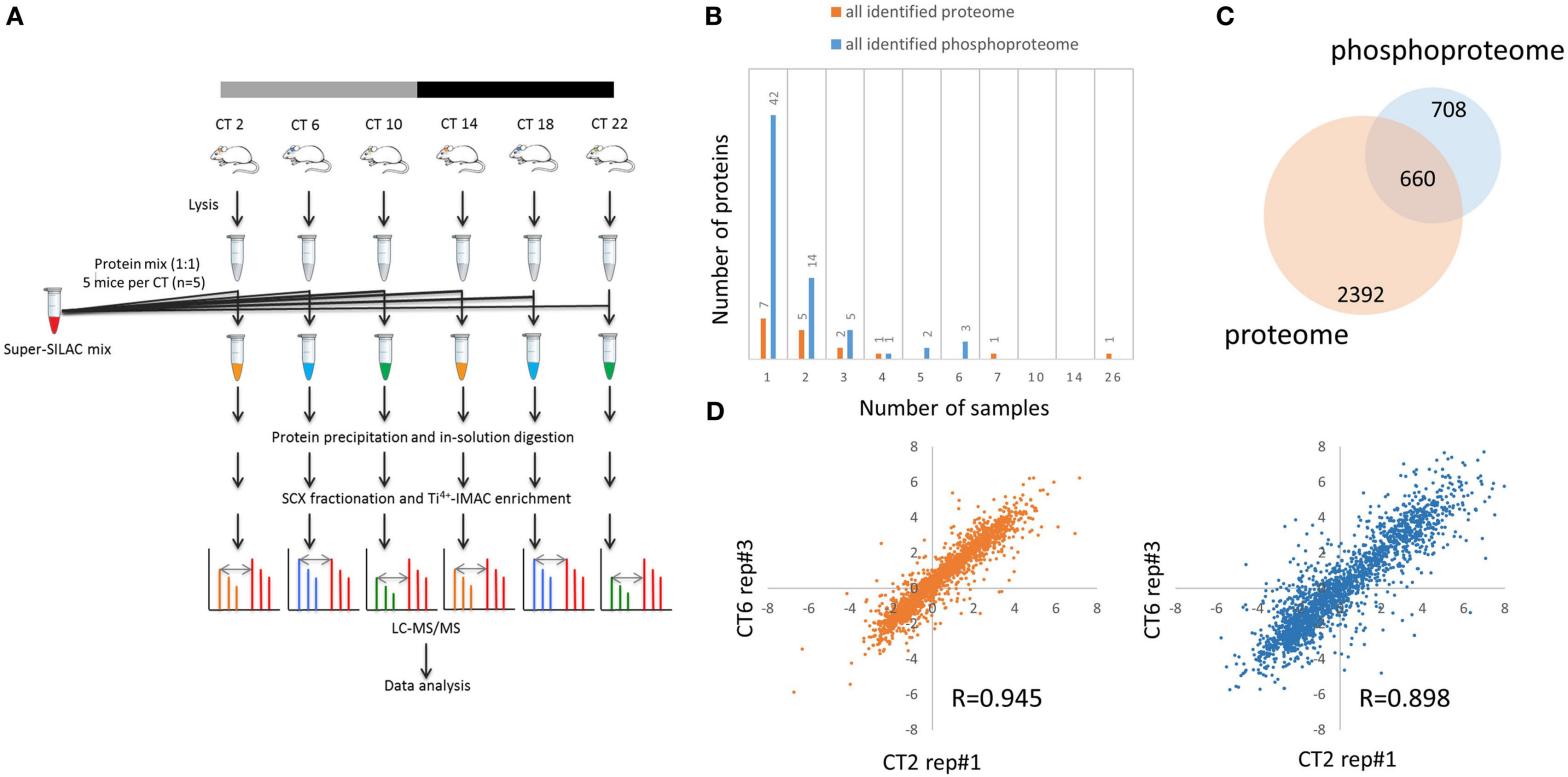
**Global proteomic and phosphoproteomic analysis of the murine hippocampus**. **(A)** Schematic overview of super-SILAC-based quantification of the murine hippocampal proteome and phosphoproteome. Proteins extracted from hippocampal tissues of individual mice [*n* = 5 per circadian time (CT); 6 CT in total] were mixed with equal quantities of protein lysates from a super-SILAC mix (five isotopically labeled cells), digested with trypsin, and processed by SCX fractionation with Ti^4+^-IMAC chromatography (phosphoproteome analysis) and without (proteome analysis). **(B)** Plot of 17 proteins and 67 phosphorylation events that were identified in the light (hippocampal tissues), but not heavy (super-SILAC mix), counterpart. *x*-axis indicates the number of hippocampal samples in which these proteins and phosphosites were detected. **(C)** Venn diagram indicates that 660 proteins were accurately quantified in both the proteome (orange) and phosphoproteome (blue) datasets, whereas 708 were found only in the phosphoproteome. **(D)** Representative scatter plots showing biological replicate measurement in proteomic and phosphoproteomic screens with a high degree of correlation among those 30 biological samples.

To explore the circadian proteome and phosphoproteome of the murine hippocampus, we stably entrained male C57BL/6J mice to a LD cycle and transferred them to constant darkness (DD) for 2 days. On day 3 of DD, hippocampal tissues were harvested from five mice at each of six time points, spaced 4 h apart (Figure 1A), to yield five independent biological replicates for each time point (5 mice per CT, *n* = 30 total mice). The proteome and phosphoproteome dynamics in the murine hippocampus over a 24-h cycle were then investigated by the state-of-art super-SILAC-based proteomics quantification method (17). This method, which results in accurate quantification, uses isotopically labeled peptides from a combination of five different SILAC-labeled cell lines with high labeling efficiency to serve as internal standards for MS-based analysis. Our super-SILAC mix included proteins from Neuro-2a (neuroblastoma), AtT-20 (pituitary), mHypoE-N38, mHypoA-2/21 (CLU-181), and mHypoA-2/28 (CLU-188) (hypothalamus) cells. “Light” hippocampal protein lysates (600 μg) were mixed with “heavy” lysates from our super-SILAC mix (120 μg of each cell line) at a 1:1 weight ratio. Following protein precipitation and tryptic digestion, 0.1 mg of the tryptic peptide mixtures were separated into five fractions for proteomic analysis, whereas 1.2 mg of resulting peptides were used for phosphopeptide enrichment analysis by Ti^4+^-IMAC chromatography (18). All fractions were analyzed by nanoLC-MS/MS on a Q-Exactive MS in a total of 300 runs.

Out of the 4,953 proteins and 9,478 phosphorylation events identified in our proteome and phosphoproteome analysis, there were 17 (0.3%) proteins and 67 (0.7%) phosphorylation events that were identified in the light samples that did not have a heavy counterpart (Figure 1B). Of these, only one protein (Ly-6/neurotoxin-like protein 1; Uniprot ID: Q9WVC2) was detected in more than half of the samples without a corresponding SILAC-labeled peak from the super-SILAC pooled standard. With a FDR of 1% at the peptide level, 6,204 of 9,478 phosphosites were classified as class I phosphorylation sites (localization probability score >0.75), with a distribution of 90.4% phosphoserine, 9.2% phosphothreonine, and 3.4% phosphotyrosine residues.

For further downstream bioinformatic analyses, we extracted from the raw dataset only the accurately quantified proteins and phosphorylation events. This resulted in more stringent, filtered datasets of 3,052 proteins (referred to as the hippocampal proteome, Table S1 in Supplementary Material) and 2,868 class I phosphorylation sites on 1,368 proteins (referred to as the hippocampal phosphoproteome, *p* > 0.75, Table S2 in Supplementary Material). Of the 1,368 phosphoproteins identified, 660 were accurately quantified in both the proteome and phosphoproteome datasets, whereas 708 were found only in the phosphoproteome, but not proteome, dataset (Figure 1C). Pairwise Pearson’s correlation analysis of 30 independent MS measurements (Tables S3 and S4 in Supplementary Material) on both hippocampal proteome and phosphoproteome data showed good reproducibility of our results (Figure 1D), with an average Pearson *r* value of 0.943 and 0.895, respectively.

### Circadian Oscillations of the Murine Hippocampal Proteome and Phosphoproteome

**Figure 2:**
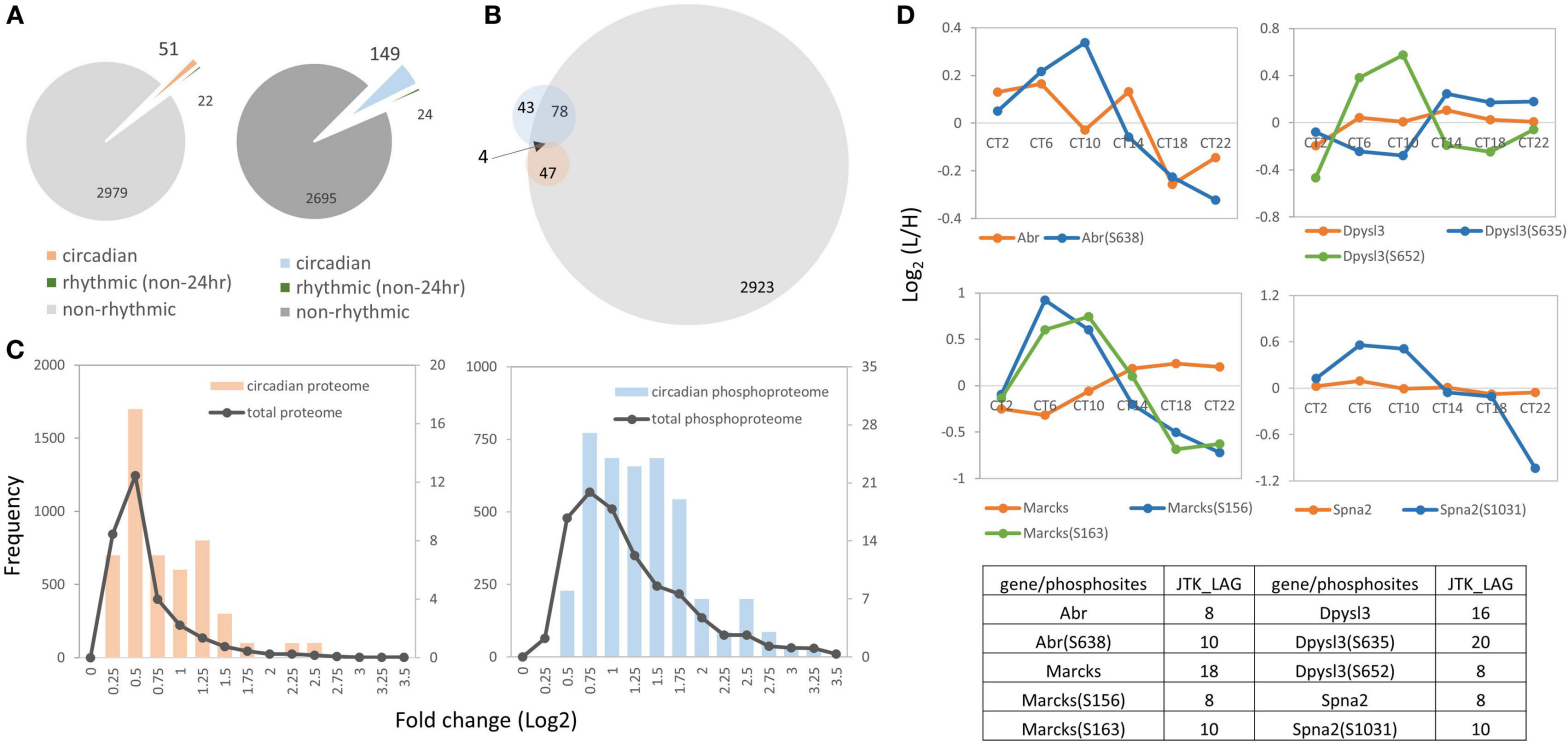
**Overview of the hippocampal circadian proteome and phosphoproteome**. **(A)** The left pie chart indicates the proportions of proteins that exhibit a circadian (orange), ultradian (8 or 12 h, green) or non-rhythmic (gray) temporal profile of abundance. The right pie chart illustrates the proportion of the phosphoproteome that exhibits a circadian (blue), ultradian (green), or non-rhythmic (dark-gray) pattern of phosphorylation (JTK_CYCLE algorithm, *p* < 0.05). **(B)** Venn diagram indicates that only four genes showed a circadian expression profile at both the protein (orange) and phosphorylation level (blue), whereas the majority of circadian phosphorylation events (97 phosphosites on 78 phosphoproteins) were non-rhythmic in terms of protein abundance (gray, hippocampal proteome). **(C)** Abundance profiles of the circadian phosphoproteome (blue) showed a higher magnitude of fluctuation than the circadian proteome (orange). Both datasets displayed a higher fold change than the total quantified dataset (gray). *x*-Axis indicates fold changes (log2 normalized ratios) of the proteome (left) and phosphoproteome (right) in a 24-h cycle. *y*-axis indicates the number of proteins or phosphopeptides from the total proteome/phosphoproteome (left *y*-axis) or from the circadian proteome/phosphoproteome (right *y*-axis). **(D)** Four proteins (*Abr, Dpysl3, Marcks*, and *Spna2*) exhibited a circadian profile at both the protein (orange) and phosphorylation (green and blue) level.

To further identify proteins and phosphoproteins that showed a circadian pattern of abundance in our murine hippocampal dataset, JTK_CYCLE algorithm (19) was employed to identify rhythmic subsets of proteins and phosphorylation events with a period of 24 h. Ultradian patterns were identified by setting the period to 8 and 12 h. As shown in Figure 2A, 51 of 3,052 (1.7%) proteins (referred to as the circadian proteome, Table S5 in Supplementary Material) and 149 of 2,868 (5.2%) phosphorylation events on 125 proteins (referred to as the circadian phosphoproteome, Table S6 in Supplementary Material) exhibited a 24-h rhythm of abundance (*p* < 0.05, JTK_CYCLE), whereas less than 1% of hippocampal proteins (22/3,052, 0.7%) and phosphorylation events (24/2,868, 0.8%) exhibited ultradian oscillations with periods of 8 or 12 h. Notably, for the majority of circadian phosphopeptides, the corresponding proteins were non-rhythmic (97 phosphorylation sites on 78 proteins, 97/149, 65.1%); only 4 genes (6 phosphosites, 6/149, 4.0%) showed a circadian expression profile at both the protein and phosphorylation level (Figure 2B).

Next, we analyzed the magnitude of the fluctuations in the circadian proteome and phosphoproteome by calculating the fold change of those cyclic subsets with the logarithmic normalized expression ratios across six CT. The mean logarithmic fold change of the circadian proteome is 0.73, whereas it is 1.26 for the circadian phosphoproteome (Figure 2C). The abundance profiles of both datasets displayed a higher fold change than the total quantified datasets (i.e., total proteome and total phosphoproteome) (Figure 2C). In cases where both the phosphorylation event and the protein abundance fluctuated in a circadian fashion, the amplitude of the phosphorylation rhythm exceeded the amplitude of the protein rhythm by approximately 40%. For example, *Abr, Dpysl3, Marcks*, and *Spna2* exhibited circadian rhythms both in their phosphorylation status and in total protein abundance, but the magnitude of the oscillation was greater for the former than it was for the latter (Figure 2D).

Collectively, our data reveal that a substantial portion of the hippocampal proteome exhibits significantly greater time-of-day oscillations at the level of phosphorylation than at the level of protein abundance, suggesting that posttranslational mechanisms play a prominent role in shaping the functions of proteins in the hippocampus.

### Phase and Site-Specific Motif Enriched Analysis of the Circadian Hippocampal Proteome and Phosphoproteome

**Figure 3:**
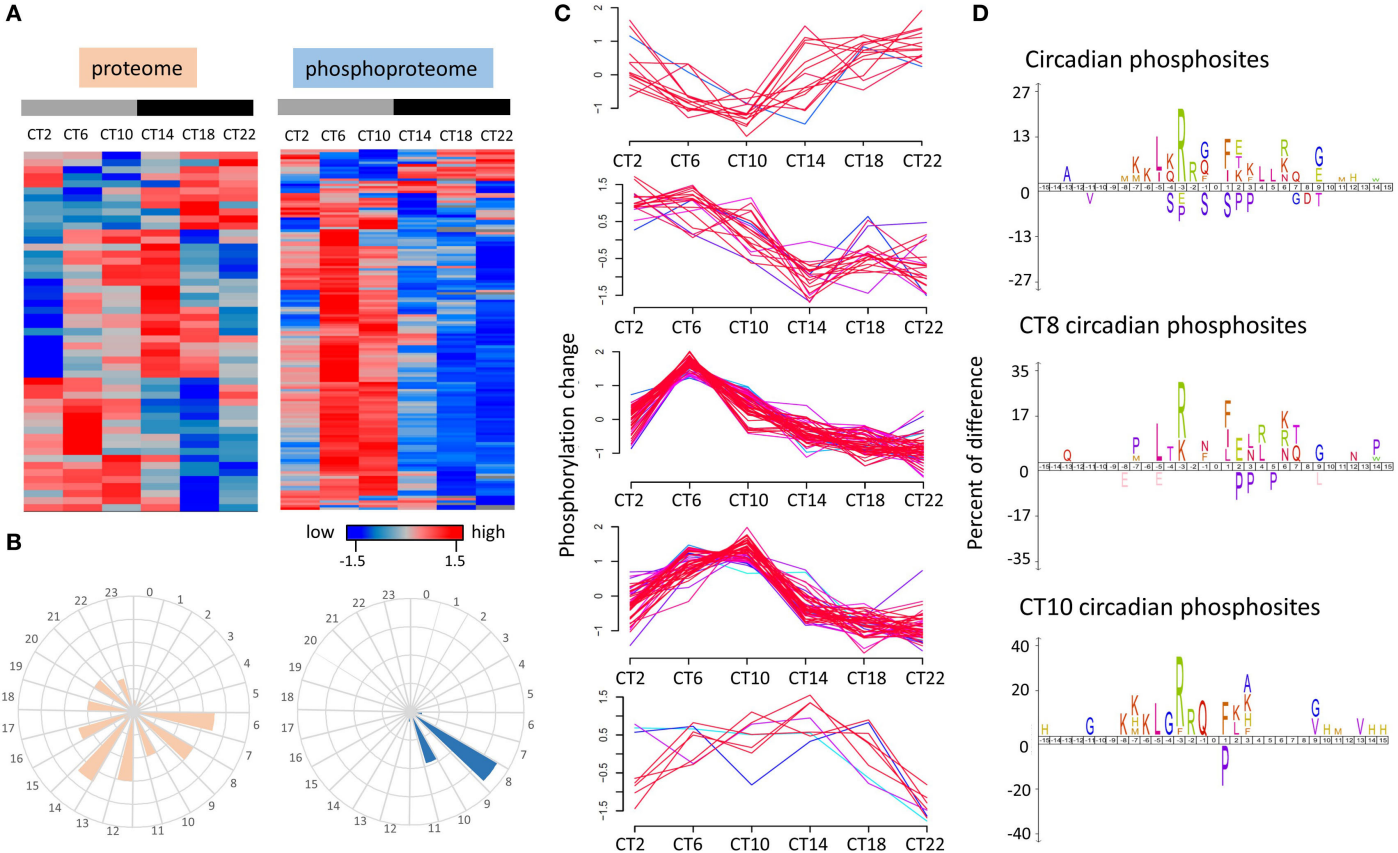
**Time-of-day profile of the circadian proteome and phosphoproteome over a 24 h cycle**. **(A)** Hierarchical clustering of the circadian proteome (left) and circadian phosphoproteome (right) in the hippocampus. **(B)** Frequency distribution of abundance phases shows that the circadian proteome is evenly distributed across the 24-h cycle, whereas the circadian phosphoproteome peaks in the mid-to-late day. **(C)** Temporal expression profile of five phosphorylation patterns that are under circadian control in the hippocampus. **(D)** Sequence logos analysis (iceLogo) of the total circadian phosphoproteome (top) as well as the subsets that peak specifically at CT8 (middle) and CT10 (bottom).

To further characterize the time-of-day-dependent proteome and phosphoproteome of the murine hippocampus, we analyzed the data by hierarchical clustering using the *z*-score normalization of the median value of normalized logarithmic expression ratios across a 24-h cycle. As shown in Figure 3A, peak times in the oscillations of the circadian proteome were evenly distributed across the 24-h cycle, whereas within the circadian phosphoproteome, the peak in abundance was largely confined to the mid-to-late day time points (CT6 and CT10). As shown in Figure 3C, unsupervised clustering analysis (fuzzy *c*-means) of the temporal profiles of cycling phosphopeptides revealed that approximately 70% of the temporal phosphorylation patterns had a marked increase in expression starting at CT2 and peaking by mid-to-late day (CT6 to CT10) and a gradual decline to trough levels in the late night (CT22). By plotting the frequency of oscillation phases of the circadian proteome and phosphoproteome, Figure 3B revealed that the phases of cycling proteins were distributed from CT6 to CT22, whereas the circadian phosphoproteins mainly peaked at CT8 (71/149 phosphosites, 47.7%) and CT10 (38/149 phosphosites, 25.5%).

Enrichment of amino acids surrounding the phosphorylated residues can be useful in revealing the broad classes of kinases that might control circadian phospho-oscillations in the hippocampus. To identify potential patterns in the enriched amino acids surrounding the phosphorylated residues of those cycling phosphoproteins, we employed iceLogo analysis (22) on the circadian phosphoproteome as well as on the peaking phosphoproteome at CT8 and CT10. Relative to all identified phosphorylation events in the murine hippocampus, circadian phosphoproteins were significantly overrepresented (*p* < 0.05) with basophilic-containing motifs, including arginine and lysine at the −2 to −7 positions, as well as hydrophobic amino acids at the −5 position (Figure 3D). Closer examination of the consensus of the cycling phosphoproteome that peaked at CT8 found a greater preference toward leucine and arginine/lysine residues at the −5 and −3 positions (Figure 3D). Phosphopeptides that peaked at CT10 exhibited an enriched motif composition that was similar to the circadian phosphoproteome with only modest differences at the C-terminal region (Figure 3D).

### Differential Distribution of the Circadian Proteome and Phosphoproteome in the Murine Hippocampus

**Figure 4:**
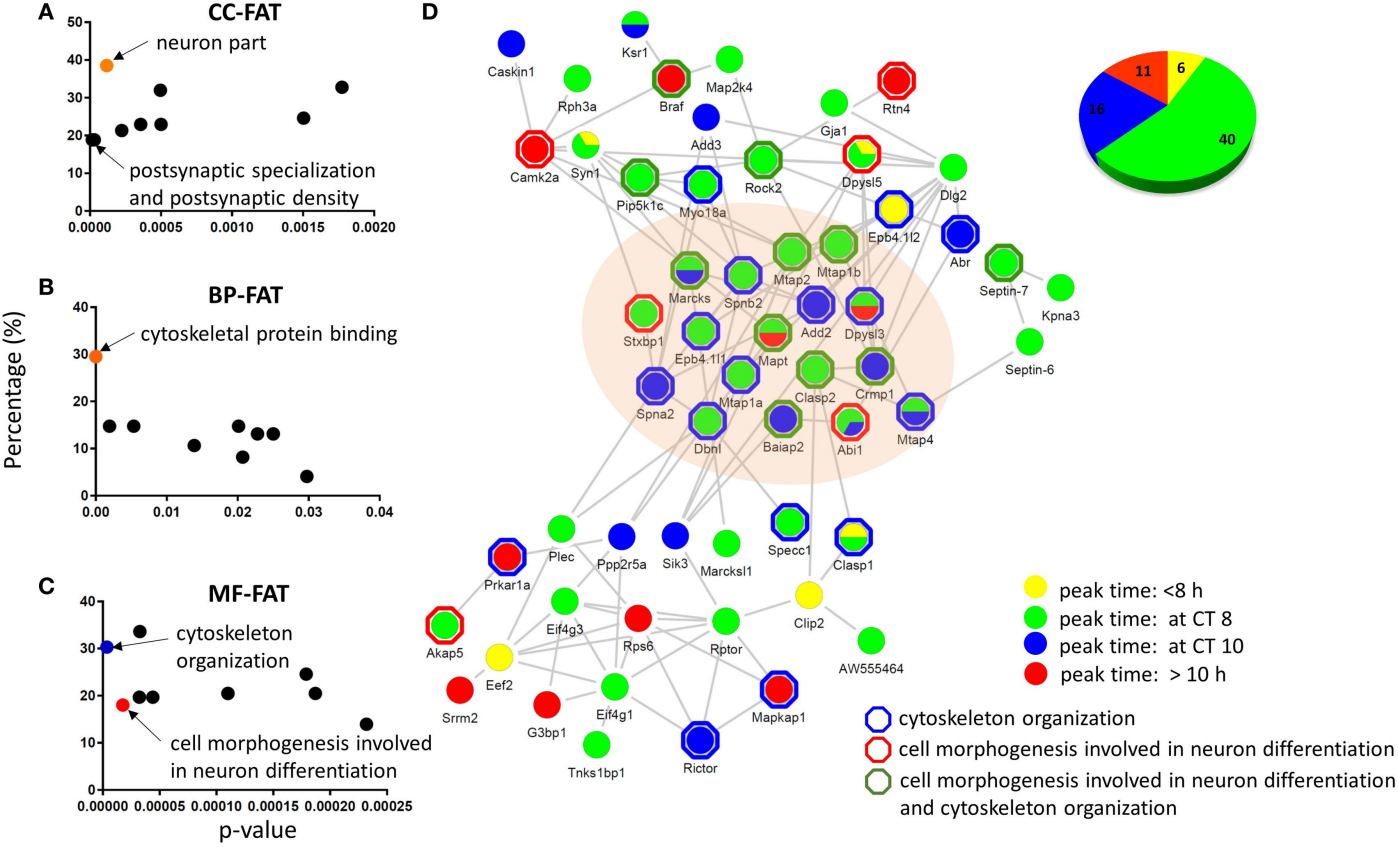
**Functional analysis and physical interaction of the circadian phosphoproteome**. Graphical distribution of overrepresented gene ontology (GO) terms of the circadian phosphoproteome against the accurately quantified phosphoproteome dataset in **(A)** cellular component, **(B)** molecular function, and **(C)** biological process by DAVID. **(D)** The largest physical interaction network of the circadian phosphoproteome in the STRING database is comprised of proteins that are described by the GO terms cell morphogenesis involved in neuron differentiation and cytoskeleton organization. Each node represents a protein that is colored according to its peak phase (yellow: between CT0 and CT8, green: at CT8, blue: at CT10, and red: between CT10 and CT24) at the phosphorylation level. Proteins outlined by a hexagon are enriched in cytoskeleton organization (blue), cell morphogenesis involved in neuron differentiation (red), or both (green) by DAVID BP-FAT analysis (Fisher’s exact test, *p* < 0.05). Inset (upper right): the pie chart indicates the proportion of phosphopeptides that belong to the four different peak phase categories.

To gain insight into the subcellular locations of, and biological processes associated with, the rhythmic phosphoproteins identified in our study, we performed GO enrichment analyses by using the bioinformatics resources available *via* the Database for Annotation, Visualization, and Integrated Discovery (DAVID, version 6.8, https://david.ncifcrf.gov/). Three available GO categories were utilized to classify the biological processes, cellular components, and molecular functions of phosphoproteins that were overrepresented in our dataset (Figures 4A–C). Relative to the accurately quantified hippocampal phosphoproteome, the circadian phosphoproteome was significantly enriched for GO-FAT cellular components that were classified as postsynaptic specialization and neuron part (Fisher’s exact test, *p* < 0.05) (Figure 4A), whereas cytoskeletal protein binding was the most highly enriched category in the GO-FAT molecular functions analysis (Figure 4B). Additionally, several metabolic pathways including cytoskeleton organization, cell morphogenesis involved in neuron differentiation, and neuron projection morphogenesis were significantly enriched in this dataset based on GO-FAT biological process analysis (Figure 4C).

To delve further into the potential biological relevance of the cycling phosphoproteome within the hippocampus, we constructed functional protein–protein interaction networks of the circadian phosphoproteome in the STRING database (Figure 4D). Out of 149 circadian phosphorylation events on 125 proteins, 57 proteins (73 phosphopeptides, 73/149 = 49.0%) exhibited a high degree of connectivity in a functional protein network (Figure 4D). This network included a relatively large number of proteins where the phosphorylation events occurred at CT8 (54.8%) and CT10 (21.9%). Notably, many of these phosphorylation events that peaked at CT8 and CT10 occurred on proteins that were classified by the GO terms cytoskeletal protein binding and/or cell morphogenesis involved in neuron differentiation.

**Figure 5:**
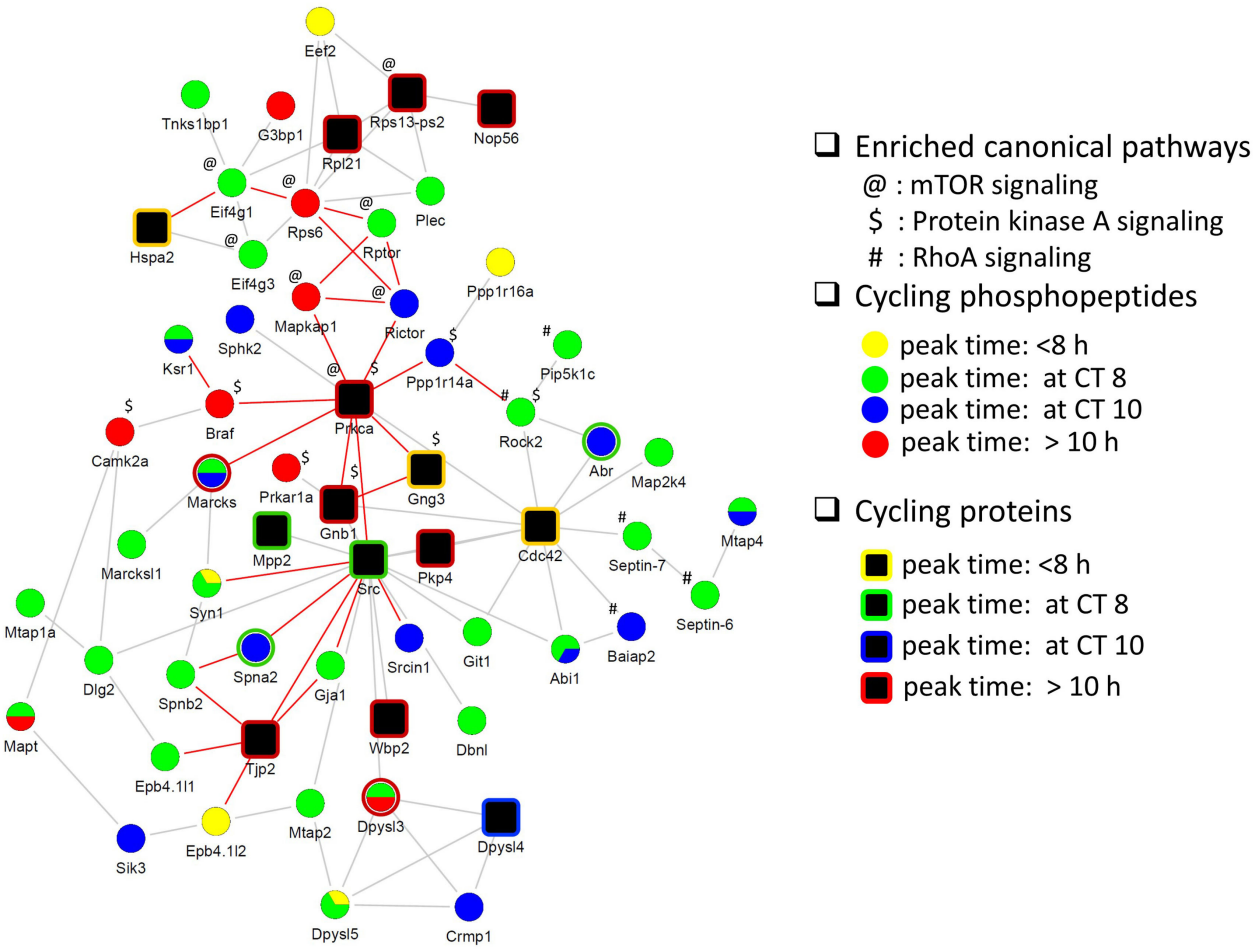
**Protein interaction network of cyclic hippocampal proteins and phosphoproteins**. Network indicating direct physical interactions between the circadian proteome (node symbol: square) and the circadian phosphoproteome (node symbol: circle). Phases of peak abundance are denoted by different colors (yellow: between CT0 and CT8, green: at CT8, blue: at CT10, and red: between CT10 and CT24). Symbols indicate proteins and/or phosphorylation events that are involved in mTOR signaling (@), protein kinase A signaling ($), and RhoA signaling (#) pathways. Proteins connected by edges (red lines) are enriched in cellular assembly and organization, cellular function, and maintenance process.

Next, we investigated the relationship between the circadian proteome and circadian phosphoproteome by constructing a direct protein interaction network using STRING, in an attempt to understand the underlying mechanisms for circadian posttranslational regulation in the hippocampus. The top functions within the largest protein interaction network that was constructed (Figure 5) were cellular assembly and organization, and cellular function and maintenance. This network included a large number of proteins that are involved in several known canonical pathways by IPA, including mTOR signaling (*p* = 3.02E−05), protein kinase A (PKA) signaling (*p* = 6.76E−05), and RhoA signaling (*p* = 4.27E−04). Prior studies have shown that phosphorylation of eIF4E, 4EBP1, rpS6, Akt, and ERK1/2 (components of the mTOR pathway) are rhythmic in the murine hippocampus and disrupting their diurnal oscillations impairs memory consolidation (26). Similar effects on memory consolidation were observed upon inhibition of PKA activity in the rat hippocampus (27).

Our collective data suggest that phosphorylation events within the hippocampus, particularly those associated with the cytoskeleton and neuronal differentiation, are under circadian regulation, peaking in the mid-to-late day. Furthermore, some of these phosphorylation events are associated with PKs that are known to be clock-controlled and/or that regulate the entrainment of the clock by light (28, 29).

### Kinase Responses and Predicted Kinase Regulators of Cycling Phosphopeptides in the Hippocampus

**Figure 6:**
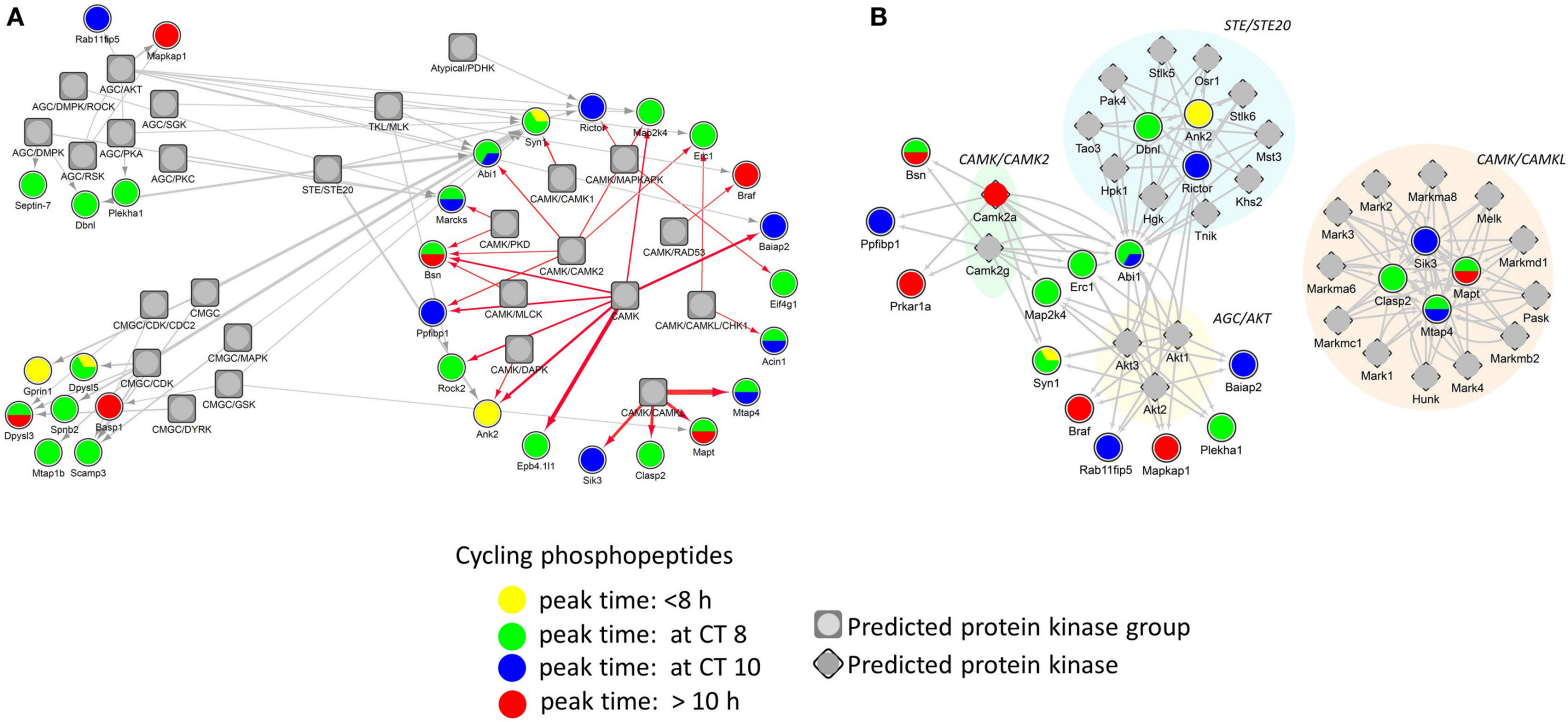
**Protein phosphorylation networks of the circadian phosphoproteome with putatively active protein kinases (PKs)**. Protein interaction networks of potential site-specific kinase–substrate relations between the circadian phosphoproteome and putative PKs by iGPS. **(A)** Top five PK groups (symbol: square) preferentially modify more phosphorylation sites on the circadian phosphoproteome. **(B)** Twenty-seven PKs (symbol: diamond) significantly modify more phosphorylation sites on the circadian phosphoproteome than the hippocampal phosphoproteome (Yates’ chi-squared test, *p* < 0.05). Each edge (line) in the network represents a circadian phosphorylation event that is mediated by the specified kinase. Phases of peak abundance of the circadian phosphoproteome (node symbol: circle) are denoted by different colors (yellow: between CT0 and CT8, green: at CT8, blue: at CT10, and red: between CT10 and CT24).

Identification of phosphorylation sites with their cognate PKs is important in understanding signal transduction within complex biological systems. In order to identify putative PKs underlying the circadian phosphoproteome, we utilized iGPS [GPS algorithm with the interaction filter, 1.0 (24)] to find kinase-specific phosphorylation sites at a high stringency level. In our systematic elucidation of ssKSRs from a circadian phosphoproteomic dataset of 149 phosphorylation events, 662 potential ssKSRs were identified among 190 PKs and 40 phosphosites (in 34 proteins), yielding a coverage rate of 26.8% (40/149). As shown in Figure 6A, top-ranking PK groups that were predicted to phosphorylate those sites belong to the AGC, CMGC, CAMK, STE, and TKL PK groups. Downstream Yates’ chi-squared test showed that, when compared to the 2,868 accurately quantified phosphoproteome, a significantly higher proportion of circadian phosphorylation events (*p* < 0.05) were predicted to be modified by 27 PKs that belong to the AKT (v-Akt murine thymoma viral oncogene), CAMK2 (Ca^2+^/calmodulin PK II), CAMKL (Ca^2+^/calmodulin PK like), or STE20 kinase families (Figure 6B).

## Discussion

Major advancements in mass spectrometric methodologies coupled with phosphopeptide enrichment strategies have allowed us to obtain an unbiased view of phosphorylation dynamics in a systematic manner (18, 30, 31). In this study, we utilize the super-SILAC-based quantitative proteomics approach as well as phophoproteomics technology to gain a first look into the circadian phosphoproteome of the murine hippocampus. Out of 3,052 proteins and 2,868 phosphopeptides that were stringently quantified, 51 (1.7%) proteins and 149 (5.2%) phosphosites exhibited a circadian expression profile. Compared to the recently published proteome studies of the SCN (4) and liver (7, 8), our proteomic and phosphoproteomic screens failed to detect any core clock proteins, likely due to their significantly lower abundance relative to the many cytoplasmic proteins, which were detected. Furthermore, although the percentage of detected circadian phosphoproteins in the hippocampus was similar to the percentage of the SCN or hepatic proteome that was rhythmic based on three previous studies (2.2% in the SCN, Chiang et al.; 6.0% in the liver, Robles et al.; 4.8% in the liver, Mauvoisin et al.), the percentage of rhythmic hippocampal proteins was markedly lower.

Protein phosphorylation and dephophorylation are highly controlled biochemical processes that respond to various intracellular and extracellular stimuli. Phosphorylation status modulates protein functions, which in turn regulate crucial biological processes and development. Wang et al. (32) showed that phosphosites on nuclear proteins in the liver were bimodally distributed at peak times in the middle of the day and the night. Robles et al. (33) discovered that phosphorylation cycles in the liver were much greater in amplitude than the fluctuations in protein abundance and markedly differed in phase when compared to the cycling proteome. In our present study, we noticed that the circadian phosphorylation events that occurred in the murine hippocampus peaked primarily at mid-to-late day (CT8 to CT10). Furthermore, the mean fold-change of the hippocampal circadian phosphoproteome was much greater than that of the hippocampal circadian proteome. The relatively high amplitude of phosphorylation rhythms is particularly noteworthy given that the hippocampus lacks intrinsic circadian rhythmicity at the level of *Per1* gene expression when cultured *ex vivo* (34). This suggests that hippocampal rhythms are either posttranscriptional or posttranslational in nature or require ongoing signals from the SCN to maintain them.

Downstream consensus motif enrichment analysis indicated that circadian hippocampal phosphopeptides, regardless of their peak phase, were good substrates for CAMK2 (R-X-X-S/T) (35) and PKD (L/I-X-R-X-X-S/T) (36), whereas those phosphopeptides that peaked specifically at CT10 possessed a favorable kinase–substrate relations with PKA (R-R-X-S/T-Y, where Y tends to be a hydrophobic residue) (37). Saraf et al. found that phosphorylation of eIF4E (Ser209), 4EBP1 (Thr37/Thr46), ERK1/2 (Thr202/Tyr204), and Akt (Ser473) in the hippocampus peaked in the mid-day to activate translation initiation and promote memory consolidation (26). Abolishing diurnal oscillations in phosphorylation of the aforementioned proteins in the hippocampus leads to a reduction in contextual memory (26). Our bioinformatics analysis also pointed to the possibility that several putative PKs belonging to the AKT, CAMK (CAMK2 and CAMKL), and STE (STE20) PK families that may play a prominent role in shaping the landscape of circadian phosphorylation events in the hippocampus. STE20 kinases are best known as members of the MAPK cascade. Eckel-Mahan et al. (38) found that daytime rhythms of MAPK activity in the hippocampus are accompanied by parallel oscillations in cAMP levels and Ras activity. In light of these previous findings, our results suggest that PKs from the CAMK and CAMKL families contribute to the circadian rhythms of protein phosphorylation within the hippocampus.

Notably, our GO enrichment analysis revealed that proteins that are categorized under postsynaptic specialization and postsynaptic density, as well as cell morphogenesis involved in neuron differentiation and cytoskeleton organization, exhibited time-of-day dependent fluctuations in their phosphorylation status. Along these lines, rats experience a rapid increase in dendritic spine density of CA1 pyramidal neurons shortly after entering the dark phase and their awake state, an effect that is mediated by various kinase pathways including MAPK/ERK, PKA, and PKC (39). Moreover, there is evidence that the sleep–wake cycle, which is coordinated by the circadian timing system, is linked to structural plasticity within the hippocampus and memory processes (40). The observed peak in hippocampal protein phosphorylation at CT8–10 suggests that the circadian timing system may be mediating anticipatory changes in proteins that are implicated in synapse function or cytoskeletal organization, in preparation for the structural changes that occur in the hippocampus shortly after wake onset.

Finally, our functional interaction network analysis of the circadian proteome and phosphoproteome indicates that signaling by mTOR, PKA, and RhoA in the hippocampus is under circadian regulation. Interestingly, PRKCA and CDC42, both of which fluctuate at the level of protein expression, appear to be major hubs that connect to rhythmically phosphorylated proteins implicated in PKA and mTOR signaling (in the case of PRKCA), and RhoA signaling (in the case of CDC42) (Figure 5). PRKCA has previously been implicated in photic entrainment of the SCN through posttranslational regulation of PER2 stability and nucleocytoplasmic trafficking (41). CDC42 belongs to the Rho family and is critical for postsynaptic structural plasticity of CA1 pyramidal neurons (42). Our study revealed that CDC42 protein levels peaked at CT6, preceding the peak in cyclic phosphorylation events that are linked to RhoA signaling by at least 2 h. The collective data from our study strongly suggest that rhythmic PTM is an important mechanism by which the circadian clock exerts temporal control of hippocampal function.

## Conclusion

Ours is the first study that investigates the circadian control of the global phosphoproteome of the murine hippocampus. Approximately 5% of detected phosphorylation events within the hippocampus oscillate in a circadian fashion and reach their peak in the mid-to-late day. In addition to this synchronicity in their peak phase, many of these phosphoproteins were associated with fundamental neuronal processes including neuronal structure. Our bioinformatics analysis also revealed putative ssKSRs within the hippocampus, thereby providing a better understanding of the mechanisms that underlie circadian regulation of hippocampal function.

## Author Contributions

C-KC, H-YC, and DF designed the experiments. C-KC, BX, NM, and WS performed the experiments. JD and HZ provided the Ti^4+^-IMAC beads. C-KC, BX, KC, JM, ZN, H-YC, and DF analyzed the data. C-KC, BX, JM, H-YC, and DF wrote the manuscript.

## Conflict of Interest Statement

The authors declare that the research was conducted in the absence of any commercial or financial relationships that could be construed as a potential conflict of interest. The reviewer MZ and handling editor declared their shared affiliation, and the handling editor states that the process nevertheless met the standards of a fair and objective review.
