# Supplementary material for: Phosphoproteome Profiling Reveals Circadian Clock Regulation of Posttranslational Modifications in the Murine Hippocampus
Source: Front Neurol. 2017 Mar 22;8:110. doi: 10.3389/fneur.2017.00110 (PMC5360755; doi:10.3389/fneur.2017.00110)
Supplement: Supplementary file 7 [file data_sheet_1.docx]

Supplementary Material

**Phosphoproteome profiling of the murine hippocampus reveals circadian clock regulation of post-translational modifications**

**Cheng-Kang Chiang, Bo Xu, Neel Mehta, Janice Mayne, Warren Y. L. Sun, Kai Cheng, Zhibin Ning, Jing Dong, Hanfa Zou, Hai-Ying Mary Cheng*, and Daniel Figeys***

**Correspondence: Hai-Ying Mary Cheng haiying.cheng@utoronto.ca. and Daniel Figeys dfigeys@uottawa.ca.**

**Supplementary Table**

Table S1.

Accurately quantified murine hippocampal proteome dataset. The list of 3052 quantified proteins resulting from the proteinGroups of MaxQuant analysis of mass spectrometry RAW files with a minimum of 15 of 30 MS measurements. Following analysis using the Perseus software, values for the ratio L/H normalized for each biological sample (30 total, n = 5 per CT, 6 different CT) are presented in columns A to AD.

Table S2.

Phosphoproteome dataset of 2868 class I phosphorylation sites (localization probability score > 0.75, column AF) sites with a minimum of 15 of 30 MS measurements in the murine hippocampus. The list of 2868 quantified phosphosites resulting from the Phospho (STY) Sites of MaxQuant and Perseus analyses. Values for the ratio L/H normalized for each biological sample (30 total, n = 5 per CT, 6 different CT) are presented in columns A to AD.

Table S3.

Pearson r correlation values of the 3052 quantified proteins in the hippocampal proteome. Pairwise Pearson's correlation analysis of 30 measurements using the hippocampal proteome dataset (3052 proteins).

Table S4.

Pairwise Pearson's correlation analysis and pearson r correlation values of the 2868 class I phosphorylation sites in the hippocampal phosphoproteome (30 measurements).

Table S5.

List of 8 h, 12 h and 24 h rhythmic proteins in the murine hippocampus. A total of 22 and 51 proteins were considered to be ultradian (8 h or 12 h rhythmic) or circadian (24 h rhythmic), respectively, based on a JTK p-value cutoff of 0.05. Column AE shows the p-value (ADJ.P). Columns AF, AG and AH indicate the period (PER), optimal phase (LAG), and amplitude (AMP) estimates, respectively, for each protein according to JTK_CYCLE algorithm analysis([1](#_ENREF_1)).

Table S6.

List of 24 and 149 phosphopeptides that displayed an ultradian (8 h or 12 h rhythmic) or circadian (24 h rhythmic) expression profile, respectively, in the murine hippocampal phosphoproteome dataset (p<0.05, JTK_CYCLE algorithm). Column AE shows the p-value (ADJ.P). Columns AF, AG and AH indicate the period (PER), optimal phase (LAG), and amplitude (AMP) estimates, respectively, for each protein according to JTK_CYCLE algorithm analysis(1).

Reference

1. Hughes ME, Hogenesch JB,Kornacker K JTK_CYCLE: an efficient nonparametric algorithm for detecting rhythmic components in genome-scale data sets. *J Biol Rhythms* (2010) **25**(5):372-80. doi: 10.1177/0748730410379711.
